# 2-[4-(Benz­yloxy)benzyl­idene]malononitrile

**DOI:** 10.1107/S1600536812020053

**Published:** 2012-05-12

**Authors:** Hai-feng Gan, Xue-wei Liu, Zheng Fang, Kai Guo

**Affiliations:** a College of Life Science and Pharmaceutical Engineering, Nanjing University of Technology, Puzhunan Road No. 30 Nanjing, Nanjing 210009, People’s Republic of China

## Abstract

In the title mol­ecule, C_17_H_12_N_2_O, the dihedral angle between the two benzene rings is 84.98 (10)°. The dicyano­ethyl­ene group is coplanar with the benzene ring to which it is bonded. No classic hydrogen bonds were found in the crystal.

## Related literature

For background information and the synthetic procedure for the title compound, see: Kharas *et al.* (2007 ▶). For a related crystal structure, see: Zhu *et al.* (2007 ▶).
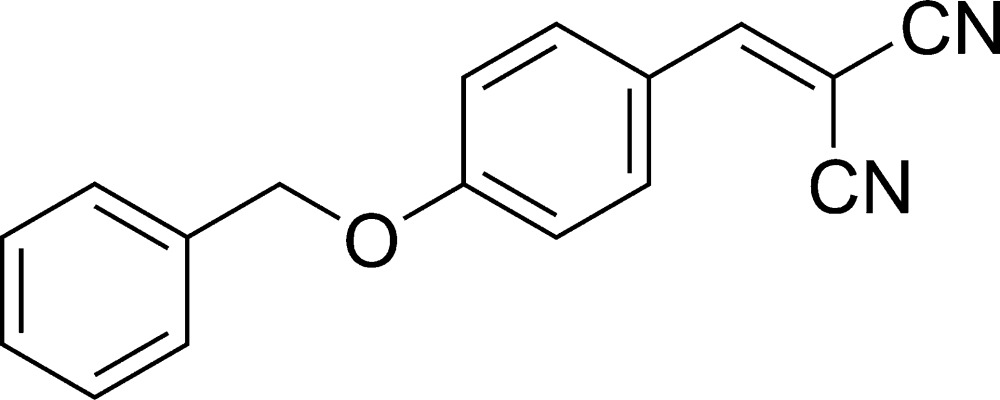


## Experimental

### 

#### Crystal data

C_17_H_12_N_2_O
*M* *_r_* = 260.29
Triclinic, 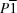

*a* = 6.8470 (14) Å
*b* = 9.6270 (19) Å
*c* = 10.544 (2) Å
α = 100.66 (3)°
β = 91.65 (3)°
γ = 94.26 (3)°
*V* = 680.5 (2) Å^3^
*Z* = 2
Mo *K*α radiation
μ = 0.08 mm^−1^
*T* = 293 K
0.30 × 0.20 × 0.10 mm

#### Data collection

Enraf–Nonius CAD-4 diffractometer
Absorption correction: ψ scan (North *et al.*, 1968 ▶) *T*_min_ = 0.976, *T*_max_ = 0.992
2722 measured reflections
2496 independent reflections
1664 reflections with *I* > 2σ(*I*)
*R*_int_ = 0.022
3 standard reflections every 200 reflections intensity decay: 1%

#### Refinement

*R*[*F* ^2^ > 2σ(*F* ^2^)] = 0.061
*wR*(*F* ^2^) = 0.176
*S* = 1.00
2496 reflections
181 parameters
H-atom parameters constrained
Δρ_max_ = 0.22 e Å^−3^
Δρ_min_ = −0.18 e Å^−3^

### 

Data collection: *CAD-4 Software* (Enraf–Nonius, 1989 ▶); cell refinement: *CAD-4 Software*; data reduction: *XCAD4* (Harms & Wocadlo, 1995 ▶); program(s) used to solve structure: *SHELXS97* (Sheldrick, 2008 ▶); program(s) used to refine structure: *SHELXL97* (Sheldrick, 2008 ▶); molecular graphics: *SHELXTL* (Sheldrick, 2008 ▶); software used to prepare material for publication: *SHELXL97*.

## Supplementary Material

Crystal structure: contains datablock(s) global, I. DOI: 10.1107/S1600536812020053/pv2535sup1.cif

Structure factors: contains datablock(s) I. DOI: 10.1107/S1600536812020053/pv2535Isup2.hkl

Supplementary material file. DOI: 10.1107/S1600536812020053/pv2535Isup3.cml

Additional supplementary materials:  crystallographic information; 3D view; checkCIF report

## Figures and Tables

**Fig. 1.**
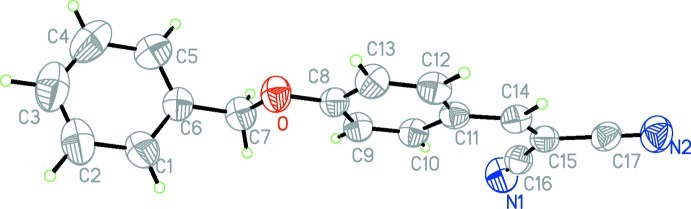
The molecular structure of the title compound with the atom numbering scheme. Displacement ellipsoids are drawn at the 50% probability level. H atoms are presented as small spheres of arbitrary radius.

**Fig. 2.**
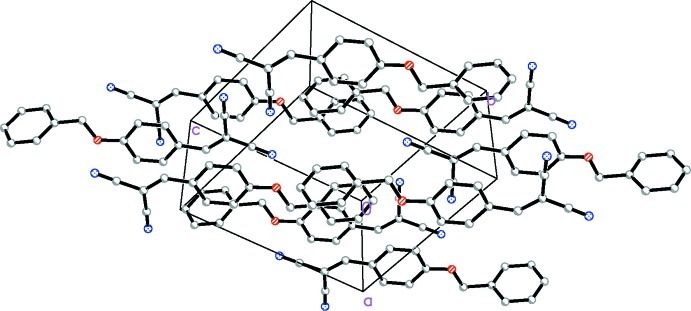
A view of the unit cell packing of the title compound.

## supplementary crystallographic information

### Comment

The synthesis of the title compound has been reoprted previously (Kharas *et al.*, 2007). It is a key intermediate in our studies of cardiovascular drugs. In this paper we report the crystal structure of the title compound.

In the title compound (Fig. 1), the dihedral angle between the benzene rings C1–C6 and C8–C13 is 84.98 (10) °. The dicyanoethylene group (N1/N2/C14–C17) is almost coplanar with the benzene ring C8–C13, with a dihedral angles between the two planes being 0.71 (8) °. The structure is devoid of any hydrogen bondinhg interactions (Fig. 2).

### Experimental

To a solution of 4-(benzyloxy)benzaldehyde (10.01 mmol, 2.12 g) and malononitrile (10.14 mmol, 0.67 g) in ethanol (20 ml) was added triethylamine (0.31 ml) and the reaction mixture was heated to 338.15 K for 3 h. The reaction mixture was cooled to room temperature and then filtered to get the title compound (2.43 g) as pure a yellow solid (Kharas *et al.*, 2007). Crystals of the title compound for X-ray diffraction were obtained by slow evaporation of an acetone solution.

### Refinement

All H atoms were positioned geometrically and refined using a riding model, with C—H = 0.93 and 0.97 Å, for aryl and methylene H-atoms, respectively. The *U*_iso_(H) were allowed at 1.2*U*_eq_(C).

### Crystal data

**Table:**

C_17_H_12_N_2_O	*Z* = 2
*M**_r_* = 260.29	*F*(000) = 272
Triclinic, *P*1	*D*_x_ = 1.270 Mg m^−^^3^
Hall symbol: -P 1	Mo *K*α radiation, λ = 0.71073 Å
*a* = 6.8470 (14) Å	Cell parameters from 25 reflections
*b* = 9.6270 (19) Å	θ = 9–13°
*c* = 10.544 (2) Å	µ = 0.08 mm^−^^1^
α = 100.66 (3)°	*T* = 293 K
β = 91.65 (3)°	Block, yellow
γ = 94.26 (3)°	0.30 × 0.20 × 0.10 mm
*V* = 680.5 (2) Å^3^	

### Data collection

**Table:**

Enraf–Nonius CAD-4 diffractometer	1664 reflections with *I* > 2σ(*I*)
Radiation source: fine-focus sealed tube	*R*_int_ = 0.022
Graphite monochromator	θ_max_ = 25.4°, θ_min_ = 2.0°
ω/2θ scans	*h* = 0→8
Absorption correction: ψ scan (North *et al.*, 1968)	*k* = −11→11
*T*_min_ = 0.976, *T*_max_ = 0.992	*l* = −12→12
2722 measured reflections	3 standard reflections every 200 reflections
2496 independent reflections	intensity decay: 1%

### Refinement

**Table:**

Refinement on *F*^2^	Primary atom site location: structure-invariant direct methods
Least-squares matrix: full	Secondary atom site location: difference Fourier map
*R*[*F*^2^ > 2σ(*F*^2^)] = 0.061	Hydrogen site location: inferred from neighbouring sites
*wR*(*F*^2^) = 0.176	H-atom parameters constrained
*S* = 1.00	*w* = 1/[σ^2^(*F*_o_^2^) + (0.1*P*)^2^ + 0.110*P*] where *P* = (*F*_o_^2^ + 2*F*_c_^2^)/3
2496 reflections	(Δ/σ)_max_ < 0.001
181 parameters	Δρ_max_ = 0.22 e Å^−^^3^
0 restraints	Δρ_min_ = −0.18 e Å^−^^3^

### Special details

**Table:**

Geometry. All e.s.d.'s (except the e.s.d. in the dihedral angle between two l.s. planes) are estimated using the full covariance matrix. The cell e.s.d.'s are taken into account individually in the estimation of e.s.d.'s in distances, angles and torsion angles; correlations between e.s.d.'s in cell parameters are only used when they are defined by crystal symmetry. An approximate (isotropic) treatment of cell e.s.d.'s is used for estimating e.s.d.'s involving l.s. planes.
Refinement. Refinement of *F*^2^ against ALL reflections. The weighted *R*-factor *wR* and goodness of fit *S* are based on *F*^2^, conventional *R*-factors *R* are based on *F*, with *F* set to zero for negative *F*^2^. The threshold expression of *F*^2^ > σ(*F*^2^) is used only for calculating *R*-factors(gt) *etc*. and is not relevant to the choice of reflections for refinement. *R*-factors based on *F*^2^ are statistically about twice as large as those based on *F*, and *R*- factors based on ALL data will be even larger.

### Fractional atomic coordinates and isotropic or equivalent isotropic displacement parameters (Å^2^)

**Table:**

	*x*	*y*	*z*	*U*_iso_*/*U*_eq_	
O	0.2390 (3)	0.75384 (16)	0.34116 (15)	0.0569 (5)	
N1	0.2812 (4)	1.3682 (3)	0.0440 (2)	0.0787 (8)	
C1	0.0380 (4)	0.6905 (3)	0.5996 (3)	0.0660 (7)	
H1A	−0.0703	0.7323	0.5729	0.079*	
N2	0.2608 (4)	1.1632 (3)	−0.3621 (2)	0.0701 (7)	
C2	0.0139 (6)	0.5918 (3)	0.6777 (3)	0.0797 (9)	
H2A	−0.1106	0.5677	0.7040	0.096*	
C3	0.1676 (7)	0.5300 (3)	0.7164 (3)	0.0851 (10)	
H3A	0.1490	0.4633	0.7694	0.102*	
C4	0.3527 (7)	0.5639 (4)	0.6788 (3)	0.0929 (11)	
H4A	0.4589	0.5201	0.7058	0.111*	
C5	0.3809 (5)	0.6648 (3)	0.5994 (3)	0.0763 (9)	
H5A	0.5057	0.6885	0.5735	0.092*	
C6	0.2224 (4)	0.7285 (2)	0.5601 (2)	0.0538 (6)	
C7	0.2449 (4)	0.8328 (3)	0.4716 (2)	0.0596 (7)	
H7A	0.1395	0.8955	0.4823	0.072*	
H7B	0.3686	0.8899	0.4911	0.072*	
C8	0.2438 (3)	0.8250 (2)	0.2424 (2)	0.0459 (6)	
C9	0.2554 (4)	0.9733 (2)	0.2554 (2)	0.0502 (6)	
H9A	0.2617	1.0303	0.3371	0.060*	
C10	0.2575 (4)	1.0339 (2)	0.1472 (2)	0.0503 (6)	
H10A	0.2657	1.1321	0.1569	0.060*	
C11	0.2476 (3)	0.9510 (2)	0.0223 (2)	0.0453 (6)	
C12	0.2362 (4)	0.8023 (2)	0.0131 (2)	0.0518 (6)	
H12A	0.2296	0.7444	−0.0682	0.062*	
C13	0.2346 (4)	0.7412 (2)	0.1198 (2)	0.0519 (6)	
H13A	0.2273	0.6430	0.1104	0.062*	
C14	0.2497 (3)	1.0026 (2)	−0.0972 (2)	0.0486 (6)	
H14A	0.2429	0.9312	−0.1703	0.058*	
C15	0.2598 (3)	1.1353 (3)	−0.1240 (2)	0.0497 (6)	
C16	0.2724 (4)	1.2637 (3)	−0.0292 (2)	0.0559 (6)	
C17	0.2608 (4)	1.1526 (3)	−0.2562 (3)	0.0538 (6)	

### Atomic displacement parameters (Å^2^)

**Table:**

	*U*^11^	*U*^22^	*U*^33^	*U*^12^	*U*^13^	*U*^23^
O	0.0747 (12)	0.0428 (9)	0.0535 (10)	0.0013 (8)	0.0064 (8)	0.0105 (7)
N1	0.103 (2)	0.0531 (14)	0.0792 (16)	0.0068 (13)	0.0117 (14)	0.0093 (13)
C1	0.0692 (19)	0.0665 (17)	0.0622 (16)	0.0038 (14)	0.0117 (14)	0.0110 (13)
N2	0.0831 (18)	0.0672 (15)	0.0647 (15)	0.0099 (12)	0.0025 (12)	0.0237 (12)
C2	0.104 (3)	0.0676 (18)	0.0684 (18)	−0.0049 (18)	0.0198 (18)	0.0170 (15)
C3	0.139 (4)	0.0565 (17)	0.0603 (18)	0.003 (2)	0.009 (2)	0.0129 (14)
C4	0.119 (3)	0.080 (2)	0.083 (2)	0.029 (2)	−0.021 (2)	0.0185 (18)
C5	0.072 (2)	0.0768 (19)	0.0817 (19)	0.0125 (16)	−0.0042 (16)	0.0169 (17)
C6	0.0641 (17)	0.0455 (13)	0.0498 (13)	0.0016 (12)	0.0016 (12)	0.0050 (11)
C7	0.0664 (17)	0.0526 (14)	0.0582 (15)	−0.0008 (12)	0.0042 (13)	0.0080 (12)
C8	0.0451 (13)	0.0403 (12)	0.0533 (13)	0.0017 (10)	0.0039 (10)	0.0122 (10)
C9	0.0575 (15)	0.0398 (12)	0.0517 (13)	0.0046 (10)	0.0032 (11)	0.0039 (10)
C10	0.0536 (15)	0.0373 (12)	0.0597 (14)	0.0038 (10)	0.0042 (11)	0.0084 (10)
C11	0.0387 (12)	0.0431 (12)	0.0546 (13)	0.0029 (10)	0.0033 (10)	0.0103 (10)
C12	0.0539 (15)	0.0441 (13)	0.0536 (14)	0.0028 (11)	0.0021 (11)	−0.0002 (11)
C13	0.0602 (16)	0.0339 (11)	0.0601 (14)	0.0003 (10)	0.0026 (12)	0.0060 (11)
C14	0.0452 (14)	0.0457 (12)	0.0540 (13)	0.0056 (10)	−0.0004 (11)	0.0068 (10)
C15	0.0439 (13)	0.0522 (14)	0.0543 (14)	0.0073 (11)	0.0045 (11)	0.0119 (11)
C16	0.0590 (16)	0.0521 (15)	0.0595 (15)	0.0054 (12)	0.0057 (12)	0.0170 (13)
C17	0.0525 (15)	0.0530 (14)	0.0597 (16)	0.0086 (11)	0.0005 (12)	0.0191 (12)

### Geometric parameters (Å, º)

**Table:**

O—C8	1.348 (3)	C7—H7B	0.9700
O—C7	1.441 (3)	C8—C13	1.388 (3)
N1—C16	1.146 (3)	C8—C9	1.405 (3)
C1—C2	1.373 (4)	C9—C10	1.374 (3)
C1—C6	1.385 (4)	C9—H9A	0.9300
C1—H1A	0.9300	C10—C11	1.405 (3)
N2—C17	1.140 (3)	C10—H10A	0.9300
C2—C3	1.337 (5)	C11—C12	1.413 (3)
C2—H2A	0.9300	C11—C14	1.437 (3)
C3—C4	1.374 (5)	C12—C13	1.362 (3)
C3—H3A	0.9300	C12—H12A	0.9300
C4—C5	1.401 (5)	C13—H13A	0.9300
C4—H4A	0.9300	C14—C15	1.356 (3)
C5—C6	1.376 (4)	C14—H14A	0.9300
C5—H5A	0.9300	C15—C16	1.434 (4)
C6—C7	1.495 (3)	C15—C17	1.434 (4)
C7—H7A	0.9700		
			
C8—O—C7	119.02 (18)	O—C8—C9	125.1 (2)
C2—C1—C6	120.6 (3)	C13—C8—C9	119.4 (2)
C2—C1—H1A	119.7	C10—C9—C8	119.9 (2)
C6—C1—H1A	119.7	C10—C9—H9A	120.1
C3—C2—C1	120.6 (3)	C8—C9—H9A	120.1
C3—C2—H2A	119.7	C9—C10—C11	121.6 (2)
C1—C2—H2A	119.7	C9—C10—H10A	119.2
C2—C3—C4	120.7 (3)	C11—C10—H10A	119.2
C2—C3—H3A	119.7	C10—C11—C12	116.8 (2)
C4—C3—H3A	119.7	C10—C11—C14	126.4 (2)
C3—C4—C5	119.7 (3)	C12—C11—C14	116.7 (2)
C3—C4—H4A	120.2	C13—C12—C11	122.0 (2)
C5—C4—H4A	120.2	C13—C12—H12A	119.0
C6—C5—C4	119.5 (3)	C11—C12—H12A	119.0
C6—C5—H5A	120.3	C12—C13—C8	120.3 (2)
C4—C5—H5A	120.3	C12—C13—H13A	119.9
C5—C6—C1	119.0 (3)	C8—C13—H13A	119.9
C5—C6—C7	121.2 (3)	C15—C14—C11	132.4 (2)
C1—C6—C7	119.8 (2)	C15—C14—H14A	113.8
O—C7—C6	107.69 (19)	C11—C14—H14A	113.8
O—C7—H7A	110.2	C14—C15—C16	125.0 (2)
C6—C7—H7A	110.2	C14—C15—C17	119.1 (2)
O—C7—H7B	110.2	C16—C15—C17	115.9 (2)
C6—C7—H7B	110.2	N1—C16—C15	178.1 (3)
H7A—C7—H7B	108.5	N2—C17—C15	178.5 (3)
O—C8—C13	115.49 (19)		
			
C6—C1—C2—C3	−0.4 (4)	C13—C8—C9—C10	0.0 (4)
C1—C2—C3—C4	0.0 (5)	C8—C9—C10—C11	0.2 (4)
C2—C3—C4—C5	0.3 (5)	C9—C10—C11—C12	−0.3 (3)
C3—C4—C5—C6	−0.1 (5)	C9—C10—C11—C14	−179.6 (2)
C4—C5—C6—C1	−0.4 (4)	C10—C11—C12—C13	0.1 (3)
C4—C5—C6—C7	−177.8 (2)	C14—C11—C12—C13	179.5 (2)
C2—C1—C6—C5	0.6 (4)	C11—C12—C13—C8	0.1 (4)
C2—C1—C6—C7	178.1 (2)	O—C8—C13—C12	179.3 (2)
C8—O—C7—C6	175.8 (2)	C9—C8—C13—C12	−0.2 (4)
C5—C6—C7—O	84.0 (3)	C10—C11—C14—C15	−0.5 (4)
C1—C6—C7—O	−93.4 (3)	C12—C11—C14—C15	−179.8 (2)
C7—O—C8—C13	−179.1 (2)	C11—C14—C15—C16	0.3 (4)
C7—O—C8—C9	0.4 (3)	C11—C14—C15—C17	179.4 (2)
O—C8—C9—C10	−179.4 (2)		
